# Influenza PB1-F2 Inhibits Avian MAVS Signaling

**DOI:** 10.3390/v12040409

**Published:** 2020-04-07

**Authors:** Yanna Xiao, Danyel Evseev, Chase A. Stevens, Adam Moghrabi, Domingo Miranzo-Navarro, Ximena Fleming-Canepa, David G. Tetrault, Katharine E. Magor

**Affiliations:** 1 Department of Medical Microbiology and Immunology, University of Alberta, Edmonton, AB T6G 2R3, Canada; yx1@ualberta.ca; 2 Department of Biological Sciences, University of Alberta, Edmonton, AB T6G 2R3, Canada; evseev@ualberta.ca (D.E.); chase3@ualberta.ca (C.A.S.); amoghrab@ualberta.ca (A.M.); miramenoparo@hotmail.com (D.M.-N.); flemingc@ualberta.ca (X.F.-C.); dtetraul@ualberta.ca (D.G.T.); 3 Li Ka Shing Institute of Virology, University of Alberta, Edmonton, AB T6G 2R3, Canada

**Keywords:** PB1-F2, NS1, type I interferon, RIG-I, duck, MAVS, innate immune response

## Abstract

RIG-I plays an essential role in the duck innate immune response to influenza infection. RIG-I engages the critical adaptor protein mitochondrial antiviral signaling (MAVS) to activate the downstream signaling pathway. The influenza A virus non-structural protein PB1-F2 interacts with MAVS in human cells to inhibit interferon production. As duck and human MAVS share only 28% amino acid similarity, it is not known whether the influenza virus can similarly inhibit MAVS signaling in avian cells. Using confocal microscopy we show that MAVS and the constitutively active N-terminal end of duck RIG-I (2CARD) co-localize in DF-1 cells, and duck MAVS is pulled down with GST-2CARD. We establish that either GST-2CARD, or duck MAVS can initiate innate signaling in chicken cells and their co-transfection augments interferon-beta promoter activity. Demonstrating the limits of cross-species interactions, duck RIG-I 2CARD initiates MAVS signaling in chicken cells, but works poorly in human cells. The D122A mutation of human 2CARD abrogates signaling by affecting MAVS engagement, and the reciprocal A120D mutation in duck 2CARD improves signaling in human cells. We show mitochondrial localization of PB1-F2 from influenza A virus strain A/Puerto Rico/8/1934 (H1N1; PR8), and its co-localization and co-immunoprecipitation with duck MAVS. PB1-F2 inhibits interferon-beta promoter activity induced by overexpression of either duck RIG-I 2CARD, full-length duck RIG-I, or duck MAVS. Finally, we show that the effect of PB1-F2 on mitochondria abrogates TRIM25-mediated ubiquitination of RIG-I CARD in both human and avian cells, while an NS1 variant from the PR8 influenza virus strain does not.

## 1. Introduction

RIG-I like receptors (RLRs) are essential pattern recognition receptors for various RNA viruses, including the influenza A virus [1]. They are DExD/H box RNA helicases comprising of three family members: Retinoic acid-inducible gene I (RIG-I), melanoma differentiation-associated gene 5 (MDA5), and laboratory of genetics and physiology 2 (LGP2) [2,3]. Both RIG-I and MDA5 can induce pro-inflammatory cytokines and type I interferon through the mitochondrial antiviral signaling (MAVS) pathway. The MAVS protein is 540 amino acids long and is composed of an N-terminal caspase activation and recruitment domain (CARD), a proline-rich region, and a C-terminal transmembrane domain, which anchors it to the membranes of mitochondria and peroxisomes [4,5], as well as mitochondrial-associated endoplasmic reticulum membranes (MAM) [6]. Activated RIG-I or MDA5 interacts with MAVS through their mutual CARD domains. The CARD domains of activated RIG-I or MDA5 bind directly to the single CARD of MAVS and nucleate a helical assembly of CARD domains from adjacent MAVS proteins, in a process called “molecular imprinting” [7]. This process promotes functional prion-like aggregates of MAVS [8], which serve as an initiation platform for a phosphorylation cascade that leads to the production of type I interferon and interferon-stimulated genes (ISGs), many of which are antiviral factors [9].

Ducks initiate an immediate and robust response to highly pathogenic avian influenza, which highlights the importance of the RIG-I pathway and downstream interferon stimulated genes (reviewed by Evseev and Magor [10]). Previously, we showed that RIG-I was associated with innate immunity [11] and was highly induced by influenza infection in ducks. In reconstitution experiments we showed duck RIG-I is capable of interacting with chicken MAVS, and in initiating downstream signaling in chicken cells [11]. The transfected duck RIG-I could also detect in vitro transcribed 5′-triphospate RNA (5′-pppRNA) or influenza infection and stimulate the production of IFN-β and some key ISGs in the naturally RIG-I-deficient chicken DF-1 cells [11,12]. RIG-I is absent in chickens [11], but MDA5 is present and MAVS signaling is functionally conserved [13,14]. Thus, duck RIG-I appears able to interact with both duck and chicken MAVS proteins. The RIG-I–MAVS interacting surfaces are not conserved between humans and ducks, but structure is conserved such that tandem T175K/T176E mutations on duck RIG-I permit interaction with human MAVS in HEK293T cells [7]. Other residues contributing to interactions between RIG-I and MAVS have not been fully explored.

Many viruses, including influenza A viruses, employ mechanisms to inhibit innate immune signaling via protein-protein interactions with or around the MAVS protein in mammals, which have been recently reviewed [15]. Non-structural protein 1 (NS1) is the most important, and best characterized IFN antagonist protein of influenza A viruses [16]. NS1 can block type I interferon signaling downstream of RIG-I [17,18,19] or by blocking RIG-I ubiquitination [20,21]. Activated RIG-I tetramers are stabilized by K63-linked polyubiquitin chains [22] synthesized by the E3 ubiquitin ligases tripartite motif-containing protein 25 (TRIM25) and RING finger protein 135 (RNF135 or Riplet) [23,24,25]. NS1 can interact directly with TRIM25 and Riplet to inhibit K63-linked ubiquitination and suppress signaling [20,21]. PB1-F2 from influenza A virus strain A/Puerto Rico/8/1934 (H1N1; PR8) also interacts with MAVS in human cells to inhibit interferon production [26].

PB1-F2 is a small accessory protein encoded by most influenza A strains in the second reading frame of the PB1 gene [27]. It is a small non-structural protein of 87–90 amino acids, translated from the 4th start codon in the +1 alternate open reading frame, located 120 bp downstream of the first start codon of PB1 gene [28]. Although not essential for viral replication, PB1-F2 may have contributed to the virulence of the Hong Kong 1997 H5N1 and the 1918 influenza strains [29]. Modulation of virulence by PB1-F2 in mammalian cells may occur through multiple mechanisms, such as promoting apoptosis, modulating innate immune responses, and exacerbating secondary bacterial infections (reviewed in [30]). Another important function of PB1-F2 is increasing the activity of the viral polymerase, which enhances replication and pathogenicity [31,32]. The large number of functions and interactions described appears to vary with context. For example, PB1-F2 proteins have been shown to inhibit type-I interferon and pro-inflammatory cytokine induction in infected mice and ferrets [33,34], but can also contribute to the induction of interferon-beta in human respiratory epithelial cells [35] and activate the NLRP3 inflammasome in mice [36]. In chickens, comparison of PB1-F2-expressing and knock-out viruses showed that PB1-F2 reduced inflammatory responses in the lungs and reduced pathogenicity in an H5N1 background [37] and prolonged viral shedding in a low-pathogenicity H9N2 background [38]. The effects of specific PB1-F2 proteins on viral pathogenesis depend in part on their variable length and amino acid polymorphisms, both of which demonstrate host–species adaptations [27,29,38,39,40,41]. Full-length PB1-F2 proteins are widely conserved in avian viruses [38]. As avian influenza A strains adapt to porcine and human hosts, the PB1-F2 proteins tend to acquire truncations [27,40].

Several studies have observed a direct interaction between the 87-amino-acid PB1-F2 of the PR8 strain and human MAVS, which dampened type I interferon signaling [26,42]. To investigate species-specificity and adaptation, we asked whether PR8 PB1-F2 would also interact with duck MAVS and interfere in the duck RIG-I signaling pathway. We found that PR8 PB1-F2 co-localizes and interacts with duck MAVS in DF-1 cells and inhibits type I interferon induction. Finally, we show that PB1-F2 abrogates TRIM25 mediated ubiquitination of RIG-I, while an NS1 variant from this strain does not.

## 2. Materials and Methods

### 2.1. Cell Culture and Transfection

DF-1 cells are spontaneously immortalized chicken embryo fibroblast cells derived from East Lansing Line (ELL-0) chicken embryos [43]. AD293T cells are derived from the commonly used HEK293T cell line and have improved cell adherence and plaque formation properties. Cells were maintained in DMEM plus 10% fetal bovine serum (FBS), and incubated at 39 °C for DF-1 cells and 37 °C for AD293T and HEK293T cells. Cells were seeded into 24-well plates at 2 × 10^5^/well or 6-well plates at 8 × 10^5^/well. The cells (70–90% confluent) were transfected 24 h later with the DNA constructs using Lipofectamine 2000^TM^ reagent (Invitrogen, Carlsbad, CA, USA) at a ratio of 1:2.5 (DNA:Lipofectamine).

### 2.2. Cloning of Duck MAVS and PR8 PB1-F2

Duck MAVS was amplified from duck cDNA and initially cloned into pCR2.1-TOPO vector (Invitrogen, Carlsbad, CA, USA). Using pCR2.1-MAVS as the template and NheI-BamHI containing primers with a V5 tag in the forward primer, we then cloned V5-tagged duck MAVS into pcDNA3.1/Hygro(+) (Thermo Fisher Scientific, Waltham, MA, USA). PB1-F2 was amplified and cloned into pcDNA3.1/Hygro(+) using cDNA reverse transcribed from A/Puerto Rico/8/1934 (H1N1) (PR8) viral RNA and BamHI-NotI containing primers with a single Flag coding sequence in the forward primer. Cloned PR8 NS1 was a kind gift from Y. Zhou. All primers used for amplification and cloning of indicated genes are listed in Supplementary Table S1. All PCR amplifications were performed with Phusion High-Fidelity DNA polymerase (NEB, Ipswich, MA, USA) or KAPA High Fidelity DNA polymerase (Roche, Basel, Switzerland) according to the manufacturer’s instructions.

### 2.3. Site Directed Mutagenesis of Human and Duck RIG-I 2CARD

The d2CARD A120D and h2CARD D122A mutants were made using QuikChange mutagenesis to introduce mutations in the wild-type 2CARD constructs previously generated [44]. Forward and reverse primers were designed according to guidelines for increased QuikChange efficiency [45]. Primers for the duck 2CARD construct contained two nucleotide replacements resulting in an A120D mutation, using preferred human codons. Primers for the human 2CARD construct contained a single nucleotide mutation to generate the D122A mutation, codon-optimized for expression in chicken cells. The GST-d2CARD A120D and GST-h2CARD D122A constructs were then created using rolling circle PCR mutagenesis using the overlapping primers and Phusion polymerase, and a DpnI digest was performed to remove the non-mutated plasmids. Sequencing confirmed the presence of the mutation and absence of any other mutations.

### 2.4. Bioinformatics

The full open reading frame of mallard duck (*Anas platyrhynchos)* MAVS is 1860 base pairs, encoding a peptide of 619 amino acids. The molecular weight of duck MAVS is predicted to be 64 kDa, using the online Expasy program (http://web.expasy.org/compute_pi/). An alignment of MAVS proteins was done using the Clustal Omega program (https://www.ebi.ac.uk/Tools/msa/clustalo/) and edited with the Boxshade server (https://embnet.vital-it.ch/software/BOX_form.html). A phylogenetic tree of MAVS was generated using MEGA7. Accession numbers for sequences are listed in Supplementary Table S2.

### 2.5. Dual Luciferase Assay

DF-1 cells were transfected with the chicken IFN-β reporter vector (150 ng/well) and *Renilla* luciferase internal control vector (10 ng/well) with dMAVS (20 ng/well), dRIG-I 2CARD (20 ng/well), full-length dRIG-I (150 ng/well), or hRIG-I 2CARD (20 ng/well) as the stimulator. In experiments with full-length dRIG-I, cells were also transfected with 500 ng/well of 5’ppp-dsRNA (RIG-I ligand, Invitrogen) 6 h after the initial transfection. AD293T cells were transfected with human IFN-β reporter vector (150 ng/well) and *Renilla* vector (10 ng/well) with hRIG-I 2CARD (20 ng/well) as the stimulator. To investigate the influence of PR8 PB1-F2 on the dMAVS signaling pathway, the Flag-PB1-F2 (PR8) construct (500 ng/well) was co-transfected with above vectors. Flag-NS1 (PR8) expression vector (500 ng/well) was included as a positive control, while vector only pcDNA3.1 (500 ng/well) or 3xFLAG (500 ng/well) were used as negative controls. Total transfected DNA quantity was always kept constant between groups using empty vector. IFN-β promoter reporter activity was measured 24 h post-transfection using the Dual Luciferase Reporter Assay System (Promega, Madison, WI, USA) as previously described. Briefly, the cells were lysed with 1× passive lysis buffer (100 μL/well) at room temperature for 15 min. Next, 20 μL of cell lysate was transferred to 1.5 mL Eppendorf tube, followed by adding 100 μL luciferase assay reagent II and 100 μL 1×Stop & Glo^®^ reagent to cell lysate, in sequence, and measuring the luciferase activities using the GloMax 20/20 Luminometer (Promega, Madison, WI, USA). The luminescence of the firefly and *Renilla* luciferases are measured separately and expressed as a ratio of firefly to *Renilla* luciferase activity.

### 2.6. Investigation of the Subcellular Distributions of Duck MAVS and PR8 PB1-F2 and Co-Localization Analyses

DF-1 cells were seeded into 6-well plates, transfected with either 2 μg (for mitochondrial co-localization) or 1 μg each (in co-transfections) of V5-MAVS, GST, GST-d2CARD, hTRIM25-V5, and/or Flag-PB1-F2 (PR8) 24 h post-seeding. In experiments where mitochondrial localization was investigated, cells were stained with 200 nM MitoTracker Red (Invitrogen, Carlsbad, CA, USA) for 30 min, 24 h post-transfection. The cells were fixed with 4% paraformaldehyde (PFA) for 20 min, and permeabilized with 0.2% Triton X-100 for 10 min. Cells were incubated with primary antibodies: mouse anti-V5 (Invitrogen, Carlsbad, CA, USA) or rabbit anti-V5 (Abcam, Cambridge, UK), mouse anti-Flag-M2 (Sigma-Aldrich, St. Louis, MO, USA), rabbit anti-GST (Sigma-Aldrich, St. Louis, MO, USA) for 1 h at room temperature, followed by one hour-incubation with the secondary antibodies (goat-anti-mouse-IgG Alexa 488 and goat-anti-rabbit-IgG Alexa 647; Thermo Fisher Scientific, Waltham, MA, USA). Nuclei were stained with 2 μg/mL Hoechst 33324. The coverslips were mounted onto microscope slides (1.0 mm thick) and examined under Confocal Microscope Zeiss LSM 710 and the captured images were processed with Zen 2011 software. Pearson’s correlation coefficient analyses were performed in ImageJ.

### 2.7. GST-Pull Down, Co-Immunoprecipitation, and Western Blotting

DF-1 or HEK293T cells were seeded into 6-well plates and co-transfected with the various indicated protein expression plasmids at 1 μg of each construct per well. Cells were collected and lysed 24 h post-transfection in 1200 μL lysis buffer (50 mM Tris pH 7.2, 150 mM NaCl, 1% [vol/vol] Triton X-100, protease inhibitor cocktail (Roche, Basel, Switzerland)) followed by centrifugation at 16,000× *g* for 10 min. GST-pull down, co-immunoprecipitation, and western blots were performed as previously described [44]. Briefly, the supernatant of cell lysate was incubated with 100 μL equilibrated glutathione-coated Sepharose 4B resin (GE Healthcare, Chicago, IL, USA) or 50 μL equilibrated mouse anti-V5 coated agarose beads (Sigma-Aldrich, St. Louis, MO, USA) overnight at 4 °C. The beads were washed three times with ice-cold lysis buffer and then boiled for 10 min in Laemmli sample buffer.

For western blots, the denatured proteins were separated by 12% SDS-PAGE and transferred to Trans-Blot Nitrocellulose transfer membrane (BioRad, Hercules, CA, USA). Membranes were blocked in 5% (w/v) skim milk/PBS for 30–60 min at room temperature, followed by incubation with the primary antibodies, mouse anti-V5 (Invitrogen, Carlsbad, CA, USA), rabbit-anti-GST (Sigma-Aldrich, St. Louis, MO, USA), mouse anti-Flag M2 (Sigma-Aldrich, St. Louis, MO, USA), and rabbit anti-V5 (Abcam, Cambridge, UK), and mouse anti-HA (GenScript, Piscataway, NJ, USA) for 1.5 h, and with the secondary antibodies, goat anti-mouse-IgG (HRP) (BioRad, Hercules, CA, USA) and goat anti-rabbit-IgG (HRP) (BioRad, Hercules, CA, USA) mixture for 1 h. To ensure that both wild type and mutant 2CARD constructs were expressed in both DF-1 and HEK293T cells, western blots were performed using rabbit anti-GST antibody and goat anti-rabbit IgG HRP-conjugated secondary, and mouse anti-actin primary antibody and goat anti-mouse IgG HRP secondary antibodies. The membranes were developed using Pierce ECL Western Blotting Substrate (Thermo Fisher Scientific, Waltham, MA, USA) and imaged using film or a digital imager (ChemiDoc, BioRad, Hercules, CA, USA).

### 2.8. Statistical Analysis

All graph bars show means ± standard deviation of three independent experiments (*n* = 3). Means were compared using one-way analysis of variance (ANOVA) with a Tukey’s multiple comparisons post-hoc test, using GraphPad Prism 6 software. *p* ≤ 0.05 was considered significant.

## 3. Results

### 3.1. Duck MAVS Has Only 28% Amino Acid Sequence Identity to Human MAVS

**Figure 1:**
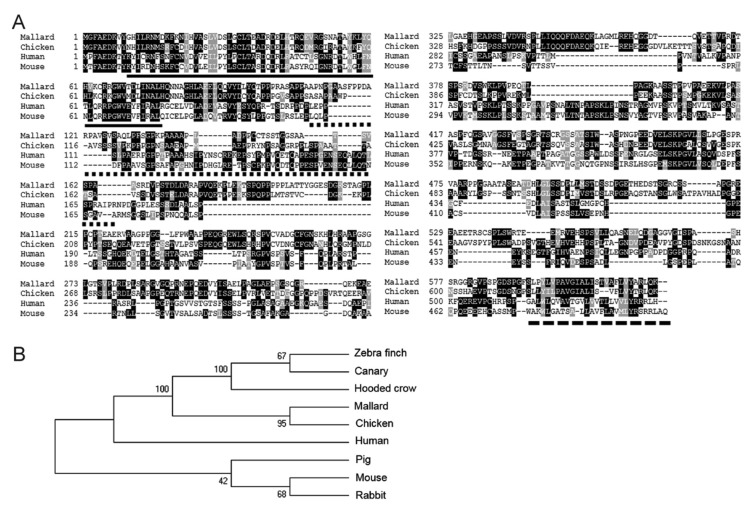
(**A**) Mitochondrial antiviral signaling (MAVS) sequences are poorly conserved between species. Amino acid alignment of duck, chicken, human, and mouse MAVS proteins was performed with Clustal Omega and edited with Boxshade. Black and grey shading indicates identical and similar amino acids (50% threshold). The solid line, dotted line, and dashed line indicate the caspase activation and recruitment domain (CARD), proline-rich region, and transmembrane domains, respectively. (**B**) Phylogenetic analysis of MAVS proteins. The bootstrap consensus tree was inferred using the Neighbor-Joining method from 1000 replicates using MEGA7. MAVS protein sequences were downloaded from the NCBI protein database (Table S2).

To compare duck MAVS to that of different species, we did an alignment of duck, chicken, human and mouse MAVS. The alignment shows that duck and chicken MAVS are quite divergent from human and mouse MAVS (Figure 1A). Duck MAVS shares low amino acid identity with chicken MAVS (58%) and human MAVS (28%). However, the CARD domain and the transmembrane (TM) domain of duck and chicken MAVS are more conserved, at 80.5% and 80% identity respectively. The proline-rich region of duck MAVS only shares 49% amino acid identity with its counterpart in chicken MAVS. Of the numerous known sites for post-translational modification of mammalian MAVS [46], only a few appear to be conserved in ducks. Sites for attachment of K48-linked ubiquitination near residues K7 and K420 in human MAVS, leading to proteasomal degradation after infection, are conserved between species [47,48]. A serine-rich cluster is present in the duck sequence near Serine 496 and contains the motif HLGLS, which resembles the consensus *p*L*x*IS motif containing (human) Serine 442 that becomes phosphorylated by IKKβ and TBK1 upon viral infection, leading to recruitment and phosphorylation of IRF3 [49]. To investigate the relationship between duck and other vertebrate MAVS, including available avian sequences, a phylogenetic tree was constructed in MEGA7. As expected, duck MAVS grouped with other avian MAVS sequences, with the closest evolutionary distance to chicken MAVS (Figure 1B).

### 3.2. Duck MAVS Localizes to Mitochondria in DF-1 Cells and Interacts with Duck RIG-I CARD Domains in the Cytoplasm

**Figure 2:**
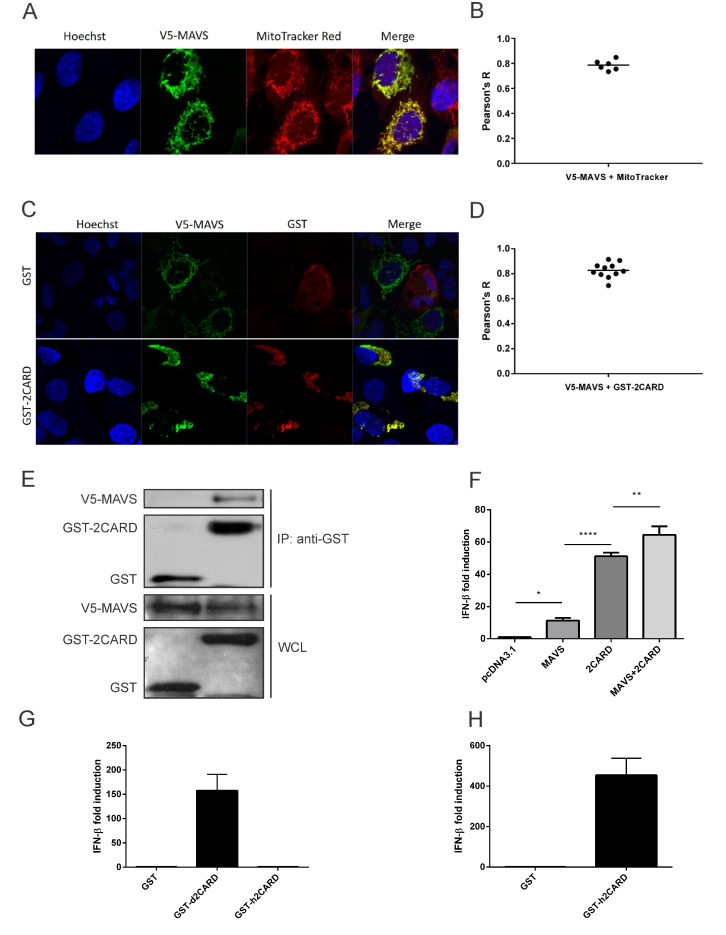
Duck MAVS localizes to mitochondria and interacts with duck RIG-I CARD domains in DF-1 cells. (**A**) DF-1 cells overexpressing duck MAVS (V5-MAVS, green) were also stained to show mitochondria (MitoTracker Red) and nuclei (blue). Original magnification X1000. (**B**) Co-localization of V5-MAVS with mitochondria was quantified using Pearson’s correlation coefficient (Pearson’s R). Bar shows mean value from 6 analyzed cells. (**C**) DF-1 cells overexpressing duck MAVS and GST-tagged duck RIG-I CARD domains (GST-2CARD) or GST alone were stained for GST (red) and V5-MAVS (green). (**D**) Co-localization of V5-MAVS with GST-2CARD was quantified using Pearson’s correlation coefficient (Pearson’s R). Bar shows mean value from 11 analyzed cells. (**E**) DF-1 cells overexpressing V5-MAVS and GST-2CARD were lysed. Clarified whole cell lysates (WCL) were subjected to GST pulldown (anti-GST) and blotted with anti-GST and anti-V5 antibodies. (**F**) Duck MAVS and RIG-I-CARD overexpression promotes chicken IFN-β promoter activity in DF-1 cells as measured by dual luciferase assay. Graph bars show mean ± standard deviation (SD) of three independent experiments (*n* = 3). Means were compared using one-way analysis of variance (ANOVA) with a Tukey’s multiple comparisons post-hoc test (*, *p* ≤ 0.05; **, *p* ≤ 0.01; ***, *p* ≤ 0.001). (**G**) In chicken DF-1 cells, duck RIG-I CARD domains (GST-d2CARD) but not human RIG-I CARD domains (GST-h2CARD) stimulate chIFN-β activity as measured by dual luciferase assay. (**H**) GST-h2CARD is functional in human AD293T cells and promoter activity of the huIFN-β reporter vector was measured by dual luciferase assay as above.

To investigate the distribution of duck MAVS in DF-1 cells, we transfected them with V5-tagged duck MAVS expression constructs and visualized them by immunofluorescence at 24h post-transfection. Visually, MAVS co-localized with the mitochondria stained with MitoTracker Red (Figure 2A). Co-localization of V5-MAVS with mitochondria was quantitatively analyzed using Pearson’s correlation coefficient (Pearson’s R). The mean Pearson’s R value was 0.79 (Figure 2B), which indicated a high signal correlation. Mitochondrial localization is consistent with mammalian MAVS [5], despite very different amino acid sequences in their transmembrane domains, which also serve as mitochondrial membrane targeting sequences.

To confirm that duck MAVS interacts with duck RIG-I-CARD, we investigated the co-localization of these two proteins with confocal microscopy. The confocal images showed that overexpressed GST on its own was diffusely distributed within the whole cytoplasm, not related to the distribution of V5-MAVS, whereas duck RIG-I CARD domains (GST-2CARD) had a similar staining pattern to duck MAVS in DF-1 cells (Figure 2C). The average Pearson’s R value for co-localization between duck MAVS and duck RIG-I-CARD was 0.83 (Figure 2D). The high signal correlation between V5-MAVS and GST-2CARD suggests that they co-localized in DF-1 cells. In co-immunoprecipitation, V5-MAVS was pulled down by GST-2CARD, but not by GST alone (Figure 2E). We confirmed that the RIG-I-CARD–MAVS interaction induces type-I interferon signaling by dual luciferase assay (Figure 2F). Compared to the pcDNA3.1 vector only, overexpression of V5-MAVS stimulated chIFN-β promoter activity approximately 8-fold in DF-1 cells, presumably through auto-assembly of overexpressed protein into the necessary helical assembly for signaling (Figure 2F). The RIG-I CARD domain fragment is free from auto-inhibition by the RIG-I C-terminal Repressor Domain and is thus constitutively active in cells. On its own, 2CARD increased promoter activity approximately 50-fold through interaction with endogenous chicken MAVS [11]. Co-expression of V5-MAVS and GST-2CARD induced the strongest chIFN-β promoter activity, suggesting a functional interaction between duck MAVS and duck RIG-I 2CARD, though it could also be additive. That RIG-I CARD domains induce interferon through functional MAVS interactions is demonstrated by the fact that duck RIG-I CARD domains (GST-d2CARD), but not human RIG-I CARD domains (GST-h2CARD), stimulated the IFN-β promoter in chicken cells, while GST-h2CARD was active in human HEK293T cells (Figure 2G and H). It is known that the RIG-I–MAVS interacting surfaces are sufficiently diverged between humans and ducks that they do not interact cross-species [7].

### 3.3. Amino Acid Residue 120 in Duck MAVS (122 in Human MAVS) Contributes to Species-Specific MAVS-CARD:: RIG-I-CARD Interactions

**Figure 3:**
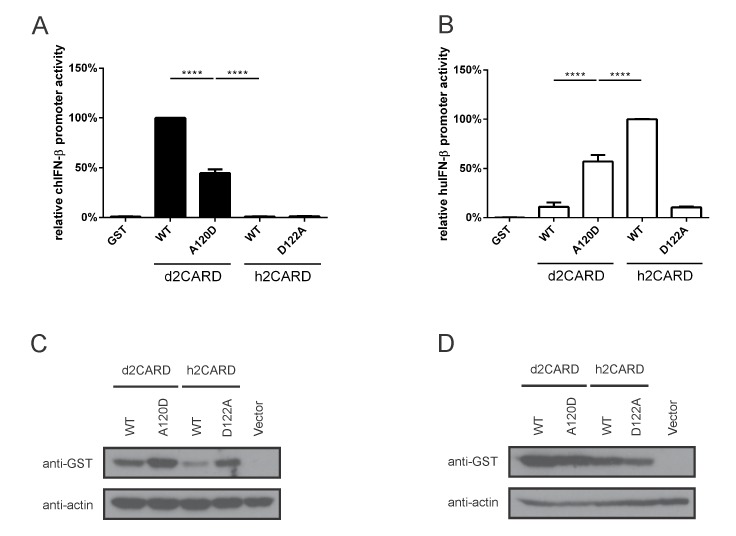
Amino acid residue 120 in duck RIG-I CARD domains (122 in human RIG-I-CARD) contributes to species-specific MAVS-CARD–RIG-I-CARD interactions. (**A**) d2CARD A120D mutation decreases IFN-β promoter activity in DF-1 cells, while h2CARD wild-type and the complementary D122A mutant remain inactive. (**B**) RIG-I d2CARD A120D mutant is more active than wild-type d2CARD in HEK293T cells, while the corresponding mutation in h2CARD nearly abrogates IFN-β promoter activity. Graph bars show mean ± SD of three independent experiments (*n* = 3). Means were compared using one-way ANOVA with a Tukey’s multiple comparisons post-hoc test (****, *p* ≤ 0.0001). Total transfected DNA quantity was always kept constant with empty vector. (**C**,**D**) All GST-2CARD proteins are expressed in DF-1 cells (**C**) and HEK293T cells (**D**). Whole cell lysates were immunoblotted with anti-GST and anti-actin antibodies.

The MAVS-CARD–RIG-I-CARD interface is poorly conserved between humans and ducks, but the overall structure is conserved such that the tandem T175K/T176E mutations on duck RIG-I permit interaction with human MAVS in HEK293T cells [7]. Other residues contributing to interactions between RIG-I and MAVS have not been fully explored. We previously noted that D122, a RIG-I residue critically involved in the human RIG-I–MAVS interaction, is replaced by A120 in ducks [44]. Notably, the mutant D122A of human RIG-I can form 2CARD tetramers, but does not engage human MAVS to initiate signaling [22]. To investigate whether altering these homologous residues would allow duck RIG-I to signal in human cells (and vice versa), we created the appropriate mutant 2CARD domains and investigated their ability to induce signaling activity compared to the wild-types in DF-1 and HEK293T cells. The wildtype d2CARD induced high chIFN-β promoter activity, while the A120D mutant d2CARD induced significantly less activity in DF-1 cells. However, neither the wildtype nor mutant h2CARD constructs induced significant promoter activity in avian cells (Figure 3A). In human cells, the RIG-I d2CARD mutation A120D increased relative human IFN-β promoter activity compared to the wildtype (Figure 3B), while the h2CARD mutation D122A almost completely abrogated relative IFN-β promoter activity in HEK293T cells. We confirmed that both sets of wild type and mutant GST-2CARD fusion proteins were expressed in DF-1 cells (Figure 3C) and HEK293T cells (Figure 3D) by western blot. All GST-2CARD proteins were expressed, but the wild type h2CARD was expressed at a lower quantity than the other proteins in DF-1 cells.

### 3.4. PR8 PB1-F2 Interacts with Duck MAVS in the Cytoplasm of DF-1 Cells and Inhibits Type-I Interferon Signaling, as It Does in Human Cells

**Figure 4:**
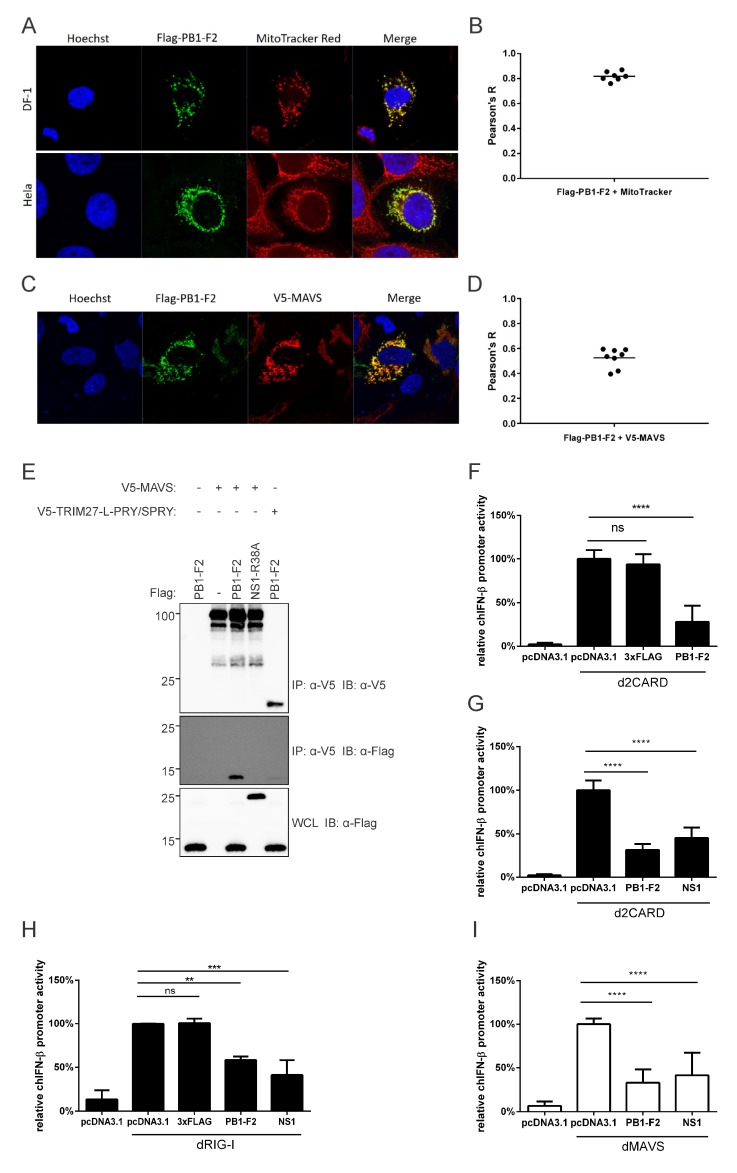
PB1-F2 co-localizes with MAVS and inhibits innate signaling activity in avian cells. (**A**) Mitochondrial localization of PB1-F2 in DF-1 and Hela cells overexpressing Flag-PB1-F2, stained for Flag-PB1-F2 (green) and mitochondria (red). Original magnification X1000. (**B**) Co-localization of PB1-F2 with MitoTracker in DF-1 cells was quantified using Pearson’s correlation coefficient (Pearson’s R). Bar shows mean value from seven analyzed cells. (**C**) DF-1 cells overexpressing Flag-PB1-F2 and V5-tagged duck MAVS (V5-MAVS) were stained with anti-Flag (green) and anti-V5 (red) antibodies. Original magnification X1000. (**D**) Co-localization of V5-MAVS with Flag-PB1-F2 was quantified using Pearson’s correlation coefficient (Pearson’s R). Bar shows mean value from eight analyzed cells. (**E**) DF-1 cells were transfected with V5-MAVS or a V5-tagged fragment of duck TRIM27-L (V5-TRIM27-L-PRY/SPRY), along with Flag-tagged PB1-F2 or Flag-tagged R38A-mutant PR8 NS1 (NS1-R38A), as indicated. Clarified whole cell lysates (WCL) were subjected to anti-V5 immunoprecipitation (IP), followed by immunoblotting (IB) with anti-V5 and anti-Flag antibodies. (**F**,**G**) PB1-F2, but not empty vector (pcDNA3.1) or the Flag tag (3xFlag), inhibits chicken IFN-β promoter activity downstream of duck RIG-I CARD domains (2CARD) in DF-1 cells, by dual luciferase assay. (**H**) PB1-F2, but not empty vector (pcDNA3.1) or the Flag tag (3xFlag), inhibits chicken IFN-β promoter activity downstream of full-length duck RIG-I (dRIG-I) in DF-1 cells, by dual luciferase assay. (**I**) PB1-F2 inhibits chicken IFN-β promoter activity downstream of duck MAVS (dMAVS) in DF-1 cells, by dual luciferase assay. Graph bars show mean ± SD of three independent experiments (*n* = 3). Means were compared using one-way ANOVA with a Tukey’s multiple comparisons post-hoc test (**, *p* ≤ 0.01; ***, *p* ≤ 0.001; ****, *p* ≤ 0.0001; ns, non-significant). Total transfected DNA quantity was always kept constant with empty vector.

PR8 PB1-F2 is targeted to the mitochondria in human cells, and interacts with mitochondrial proteins, like MAVS and Tom40 (a major mitochondrial outer membrane import channel) [50]. To examine the distribution of PR8 PB1-F2 in avian cells, we transfected a Flag-tagged PB1-F2 expression vector into DF-1 cells and performed confocal microscopy and co-localization analysis. The distribution of PB1-F2 in HeLa cells served as the positive control. The confocal images show that PB1-F2 had a clearly similar staining pattern to the MitoTracker dye in both DF-1 cells and HeLa cells (Figure 4A). The co-localization of PR8 PB1-F2 with mitochondria in DF-1 cells was also quantitatively analyzed, with a mean Pearson’s R value of 0.82 (Figure 4B). The high signal correlation between PB1-F2 and MitoTracker staining indicated that PR8 PB1-F2 is also distributed on mitochondria of DF-1 cells, consistent with its distribution in human cells. To determine whether PB1-F2 interacts with mallard duck MAVS, we co-transfected DF-1 cells with Flag-tagged PB1-F2, and V5-MAVS. PB1-F2 had a similar staining pattern to duck MAVS (Figure 4C). The average Pearsons’s R value of 0.67 indicated moderate co-localization (Figure 4D). PB1-F2 co-immunoprecipitated with V5-MAVS, but not with another V5-tagged duck protein, the PRY/SPRY domain fragment of duck TRIM27-L [51], suggesting a specific interaction (Figure 4E).

To investigate whether PR8 PB1-F2 inhibits interferon induction through MAVS, as it does in human cells [52], we performed dual luciferase reporter assays. PB1-F2 inhibited chIFN-β promoter activity downstream of duck RIG-I CARD domains (Figure 4F,G) and downstream of full-length duck RIG-I (Figure 4H). Similarly, PR8 PB1-F2 inhibited interferon promoter activity downstream of duck MAVS by over 50% (Figure 4I). Wild-type PR8 NS1 also inhibited interferon signaling in both cases, while the empty expression vector and the Flag tag on their own had no effect.

### 3.5. PR8 PB1-F2 Inhibits TRIM25-Mediated Ubiquitination of Both Human and Duck RIG-I CARD Domains

**Figure 5:**
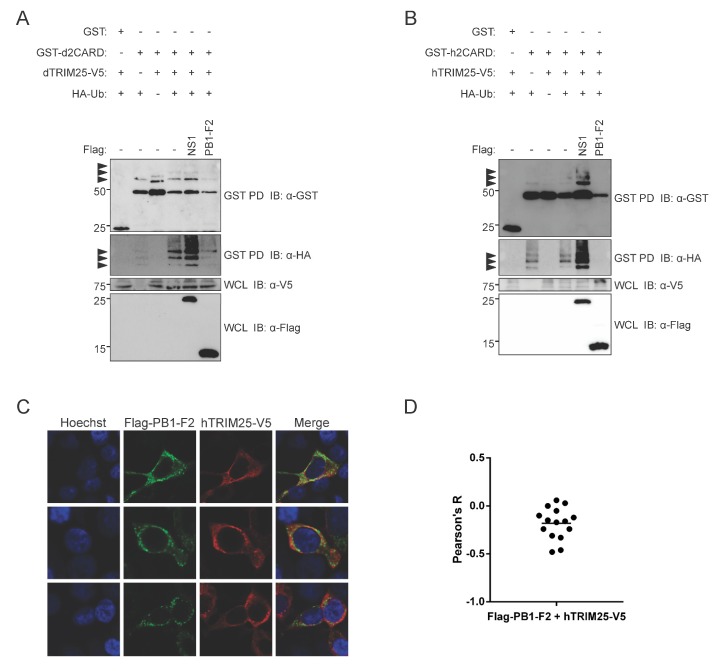
PB1-F2 inhibits TRIM25-mediated RIG-I-CARD ubiquitination. (**A**) DF-1 cells were transfected with GST-tagged duck RIG-I CARD domains (GST-d2CARD) or empty GST vector, together with V5-tagged duck TRIM25 (dTRIM25-V5), HA-tagged ubiquitin (HA-Ub), and the indicated Flag-tagged proteins (NS1 or PB1-F2). 24 h post-transfection, clarified whole cell lysates (WCL) were subjected to GST pulldown (GST PD), followed by immunoblotting (IB) with anti-GST, anti-HA, anti-V5, and anti-Flag antibodies. Arrowheads indicate ubiquitinated bands. (**B**) HEK293T cells were transfected with GST-tagged human RIG-I CARD domains (GST-h2CARD) or empty GST vector, together with V5-tagged human TRIM25 (hTRIM25-V5), HA-tagged ubiquitin (HA-Ub), and the indicated Flag-tagged proteins (NS1 or PB1-F2). GST pulldown and immunoblotting were performed as above (**C**) PR8 PB1-F2 localizes to cytoplasmic puncta that partially exclude human TRIM25. HEK293T cells overexpressing hTRIM25-V5 and Flag-PR8-PB1-F2 proteins were fixed, stained, and imaged using confocal microscopy. Panels show staining with Hoechst 33324 (nuclei, blue), anti-Flag antibodies (green), anti-V5 antibodies (red), and a merged image. Original magnification X1000. (**D**) Co-localization of Flag-PR8-PB1-F2 with hTRIM25-V5 was quantified using Pearson’s correlation coefficient (Pearson’s R). Bar shows mean value from 15 analyzed cells.

To determine whether the mechanism by which PB1-F2 interferes with innate immune signaling involves ubiquitination of RIG-I 2CARD, as previously shown for influenza A NS1, we examined the effect of PB1-F2 on TRIM25-mediated ubiquitination of RIG-I-CARD. We transfected DF-1 cells with GST-d2CARD, dTRIM25-V5, and HA-tagged ubiquitin to visually assess the ubiquitination level of the CARD domains. We found that PB1-F2 co-expression visibly decreased d2CARD ubiquitination by duck TRIM25, while the PR8 NS1 variant that we used did not (Figure 5A). We saw a similar pattern in HEK293T cells expressing GST-h2CARD, hTRIM25-V5, and HA-ubiquitin, where PB1-F2 and not a PR8 NS1 variant inhibited TRIM25-mediated ubiquitination (Figure 5B). PB1-F2 also inhibited GST-h2CARD ubiquitination by endogenous TRIM25 as well (Supplementary Figure S1). We examined the subcellular distribution of PB1-F2 and hTRIM25-V5 in HEK293T cells with confocal microscopy. PB1-F2 had a punctate distribution in the cytoplasm, and the large puncta appeared to exclude the otherwise diffusely cytoplasmic hTRIM25-V5 (Figure 5C). A slightly negative average Pearson’s coefficient indicated lack of co-localization (Figure 5D).

## 4. Discussion

Duck MAVS localizes to the mitochondria of chicken DF-1 cells and overexpression induces IFN-β reporter activity, indicating duck MAVS function is conserved in chicken cells, even though it shares a low amino acid identity with chicken MAVS. PR8 PB1-F2 interacts with duck MAVS in the cytoplasm of DF-1 cells and inhibits interferon-β promoter activity downstream of both duck RIG-I and duck MAVS.

Signaling of duck MAVS was recently shown in duck embryonic fibroblast cells [53], however, transfections were much more reproducible in DF-1 cells. Duck MAVS co-localized and co-precipitated with duck RIG-I CARD domains in DF-1 cells. Their co-expression induced chicken IFN-β promoter activity more strongly than either protein on its own, suggesting a productive interaction. In contrast, human RIG-I CARD domains did not induce IFN-β in avian cells, suggesting that h2CARD cannot interact with chicken MAVS. The inverse is also true [7]. This is partly due to an amino acid polymorphism on RIG-I-CARD that lies at the interface between MAVS and RIG-I (A120 in duck MAVS, homologous to D122 in human MAVS) that we previously noted [45]. A mutation of the alanine residue in duck 2CARD to aspartate partially restored its activity in human cells, while decreasing its activity in avian cells. Activity was only partially restored because of the presence of other polymorphisms at the interface. Previously, we showed that tandem mutation of the residues T175K/T176E in d2CARD allowed it to engage the appropriate residues on the CARD of human MAVS and restore filament formation and immunostimulatory activity [7]. The additive effects of all three mutations could be interesting to investigate.

Using the reconstituted RIG-I–MAVS signaling pathway in chicken DF-1 cells, we investigated the function of PR8 PB1-F2. First, we observed the mitochondrial distribution of PB1-F2, consistent with its localization in HeLa cells [28,52]. We also demonstrated co-localization and immunoprecipitation with duck MAVS, suggesting that the interaction with PB1-F2 is conserved. PB1-F2 has been shown to bind to the C-terminal region containing the transmembrane domain of mammalian MAVS [42], which is poorly conserved between avian and mammalian orthologues. Remarkably, although duck MAVS shares only 28% identity with human MAVS, PR8 PB1-F2 appears to interact with both proteins. The C-terminal region of MAVS includes the transmembrane domain and a conserved phosphorylation motif that allows MAVS to recruit IRF3 [49]. In their investigation of PR8 PB1-F2 and human MAVS, Varga et al. [43] proposed that PB1-F2 could interfere with the correct formation of MAVS-associated signaling complexes, but also showed that mitochondrial membrane potential was disrupted by PB1-F2. Mitochondrial membrane potential is necessary for proper MAVS signaling [54]. Varga et al. [43] suggested that both mechanisms, direct disruption of MAVS complex formation by PB1-F2 and disruption through membrane potential loss, could be contributing to signaling suppression. Another group confirmed that PR8 PB1-F2 disrupts mitochondrial membrane potential and observed translocation of PB1-F2 into the mitochondrial inner membrane space [50]. Both the interaction with MAVS and the disruption of membrane potential depend on the C-terminus of PB1-F2. It is unclear in our results which of these two mechanisms, and to what extent, contribute to the interferon inhibition in the duck RIG-I pathway. PB1-F2 decreased signaling stimulated by overexpression of either duck MAVS or duck RIG-I-CARD. Therefore, if interference occurs at the level of MAVS, this suggests that PB1-F2 can interact with both duck and chicken MAVS.

Unexpectedly, we observed that expression of PB1-F2 inhibits TRIM25-mediated ubiquitination of RIG-I-CARD, in human and avian cells. PB1-F2 may directly block ubiquitination by direct interaction with TRIM25, but this seems unlikely given their lack of co-localization in HEK293T cells. The fact that PB1-F2 aggregates in the cell cytoplasm exclude TRIM25 suggests that PB1-F2 could perhaps sequester RIG-I CARD domains away from TRIM25. No mitochondrial staining was done on the TRIM25 co-expression slides so it is unclear if PB1-F2 mitochondrial co-localization is disrupted. Alternatively, loss of ubiquitination may be a consequence of negative feedback from disrupted MAVS or the loss of mitochondrial membrane potential. RIG-I signaling is tightly regulated in cells through a complex network of positive and negative feedback mechanisms that involve several different de-ubiquitinating enzymes (reviewed in [55]). Indirect and reciprocal negative regulatory mechanisms have also been previously described [56,57], including the induction of autophagy through MAVS [58]. PR8 NS1 was expected to inhibit human TRIM25 mediated ubiquitination [24], however, we observed increased ubiquitination. We suspect this result is consistent with different strains of NS1 having a different capacity to affect TRIM25-mediated ubiquitination [21]. Several natural variants of PR8 NS1 exist, with amino acid polymorphisms that may affect the ability of individual variants to inhibit TRIM25-mediated ubiquitination. We are currently investigating the consequences of these polymorphisms in this context. Our data fit with a model in which PB1-F2 interferes with MAVS signal transduction potentially through loss of mitochondrial membrane potential, which also abrogates TRIM25-mediated ubiquitination of RIG-I CARD domains.

## 5. Conclusions

Here we demonstrate that the function of MAVS in the RIG-I signaling pathway is conserved in ducks and that PR8 PB1-F2 may interfere in this signaling pathway as it does in human cells. In DF-1 cells, PB1-F2 may perturb MAVS signaling complex assembly, mitochondrial membrane potential, RIG-I ubiquitination, or a combination of these things. In this context, PR8 PB1-F2 demonstrates a conservation of function across diverse host species.

## Supplementary Materials

The following are available online at https://www.mdpi.com/1999-4915/12/4/409/s1, Table S1: Primer list, Table S2: Accession numbers for MAVS proteins, Figure S1: PB1-F2 inhibits human RIG-I-CARD ubiquitination.
