# Supplementary material for: Influenza PB1-F2 Inhibits Avian MAVS Signaling
Source: Viruses. 2020 Apr 7;12(4):409. doi: 10.3390/v12040409 (PMC7232376; doi:10.3390/v12040409)
Supplement: Supplementary file 1 [file viruses-12-00409-s001.pdf]

viruses

MDPI

Table S1. Primer list

Name	Sequence (5' to 3')	Application
duMAVS F	ATGGGTTTCGCGGAGGACAAG	duMAVS cDNA amplification
duMAVS R	CTATTTCTGCAGCCGGGCGTAC	duMAVS cDNA amplification
NheI-V5-duMAVS F	GCCTGCTAGCATGGGCAAGCCCATCCCCAACCCCTTGCTG GCTTGGACTCCACCGGTTTCGCGGAGGACAAGGTG	V5-duMAVS amplification
duMAVS-BamH1 R	CGGAGGATCCCTACTATTTCTGCAGCCGGGCGTAC	V5-duMAVS amplification
BamHI-Flag-PB1-F2 F	GGATCCGCCACCGATTATAAAGATGATGATGATAAAGGA ATGGGACAGGAACAGGATACA	Flag-PB1-F2 (PR8) amplification
PB1-F2-NotI R	GCGGCCGCCCTACTCGTGTTTGCTGAACAAC	Flag-PB1-F2 (PR8) amplification
h2CARD D122A F	AACCAGAATTATCCCAACCGCTATCATTTCTGATCTGTCT GAATGTTTAATTAATCAGG	Mutagenesis h2CARD D122A
h2CARD D122A R	CAGACAGATCAGAAATGATAGCGGTTGGGATAATTCTGG TTTTAAATTCTGG	Mutagenesis h2CARD D122A
d2CARD D120A F	GTTAGAAGTCGACCCAGTAGATCTCATTCCCTATATAAGC ACATGCCTGATAGAC	Mutagenesis d2CARD A120D
d2CARD D120A R	GTGCTTATATAGGGAATGAGATCTACTGGGTCGACTTCTA ACATTGTTGCCTCTATCCG	Mutagenesis d2CARD A120D

Table S2. Accession numbers for MAVS proteins

Number	Common name	Accession #
1	human	NP_065797.2
2	mouse	NP_001193314.1
3	pig	NP_001090898.1
4	rabbit	XP_017197245.1
5	duck	XP_021130364.1
6	chicken	NP_001012911.1
7	canary	XP_018778718.1
8	hooded crow	XP_010400416.1
9	zebra finch	XP_002188030.1

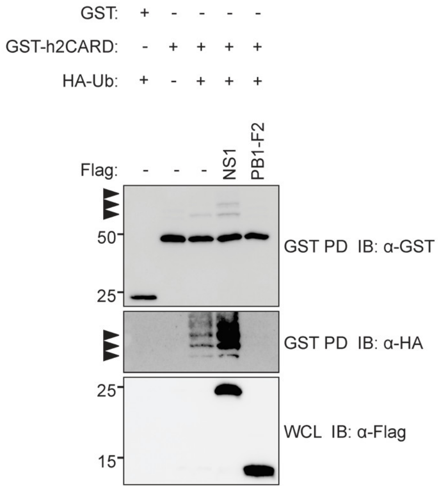

Figure S1. PB1-F2 inhibits human RIG-I-CARD ubiquitination. HEK293T cells were transfected with GST-tagged RIG-I CARD domains (GST-h2CARD) or empty GST vector, together with HA-tagged ubiquitin (HA-Ub) and the indicated Flag-tagged proteins (NS1 or PB1-F2). Clarified whole cell lysates (WCL) were subjected to GST pulldown (GST PD) 24 h post-transfection, followed by immunoblotting (IB) with anti-GST, anti-HA, anti-V5, and anti-Flag antibodies. Arrowheads indicate ubiquitinated bands.
